# Transvaginal evisceration progressing to peritonitis in the emergency department: a case report

**DOI:** 10.1186/1865-1380-4-66

**Published:** 2011-10-13

**Authors:** Luan Lawson, Leigh Patterson, Kelly Carter

**Affiliations:** 1 Department of Emergency Medicine, Brody School of Medicine, East Carolina University, 600 Moye Boulevard, Greenville, NC 27834 USA; 2 Johnston Memorial Hospital, 351 Court Street North East, Abingdon, VA 24210 USA

## Abstract

**Background:**

Abdominal pain is a common complaint among emergency department patients, making it essential to identify those with life-threatening etiologies. We report on the rare finding of atraumatic transvaginal bowel evisceration in a patient presenting to the emergency department with the primary complaint of abdominal pain.

**Case Description:**

A 63-year-old female presented ambulatory to the emergency department with abdominal pain and foreign body sensation in her vagina after coughing. Physical exam demonstrated evisceration of her small bowel through her vagina. During her clinical course, she rapidly deteriorated from appearing well without abdominal tenderness to hypotensive with frank peritonitis.

**Conclusion:**

This case demonstrates the need to perform a thorough physical exam on all patients with abdominal pain and details the management of vaginal evisceration. This case also highlights the difficulty of appropriate triage for patients with complaints not easily assessed in triage. In an era of emergency department crowding, emergency physicians should reevaluate nursing education on triaging abdominal pain to prevent delays in caring for well-appearing patients who have underlying life-threatening illnesses.

## Background

Abdominal pain is the most common presentation to US emergency departments (ED) and accounts for 6.8% of all visits [1]. Identifying those patients with abdominal pain who are at risk for acute decompensation is essential. Evisceration of bowel through the vagina is a rarely reported complication of a hysterectomy. It is more commonly associated with trauma and conditions that increase intra-abdominal pressure, including heavy lifting, coughing or straining [2]. Much of the literature on this topic is available in obstetrics and gynecology journals [2-4]. We report this rare finding in a patient presenting to the emergency department with the common chief complaint of abdominal pain.

## Case description

A 63-year-old G2P2 female presented ambulatory to the ED with the chief complaint of abdominal pain, described further to the triage nurse as abdominal cramping and a mass in her vagina. The patient described that she had had a "bulge" in her vagina for the past 2 years and was currently being treated by her gynecologist for an enterocele with estrogen cream. Elective surgical repair of an enterocele was planned. She complained to the triage nurse of abdominal pain intermittently for the preceding 1 week. She stated that when she coughed something protruded from her vagina and she believed that her rectum had prolapsed. She was initially triaged to the lower acuity area of the emergency department, but due to worsening pain, she was brought back to a room on the acute care side approximately 30 min after her arrival. She described working in her garden when she coughed, experiencing a "bulge" extending through and out of her vagina. According to the patient, this "bulge" had been worsening for 2 months but had never extended past her labia. The patient complained of severe cramping in the left lower quadrant of her abdomen, but denied any diffuse abdominal pain. Her past medical history was significant for hypertension and breast cancer treated with surgery and chemotherapy without radiation. The patient did not have a history of vaginal or vulvar cancer. Her surgical history was significant for mastectomy and breast reconstruction, laparoscopic-assisted vaginal hysterectomy and bilateral oopherectomy (5 years previously), and pubovaginal sling (4 years previously).

**Figure 1:**
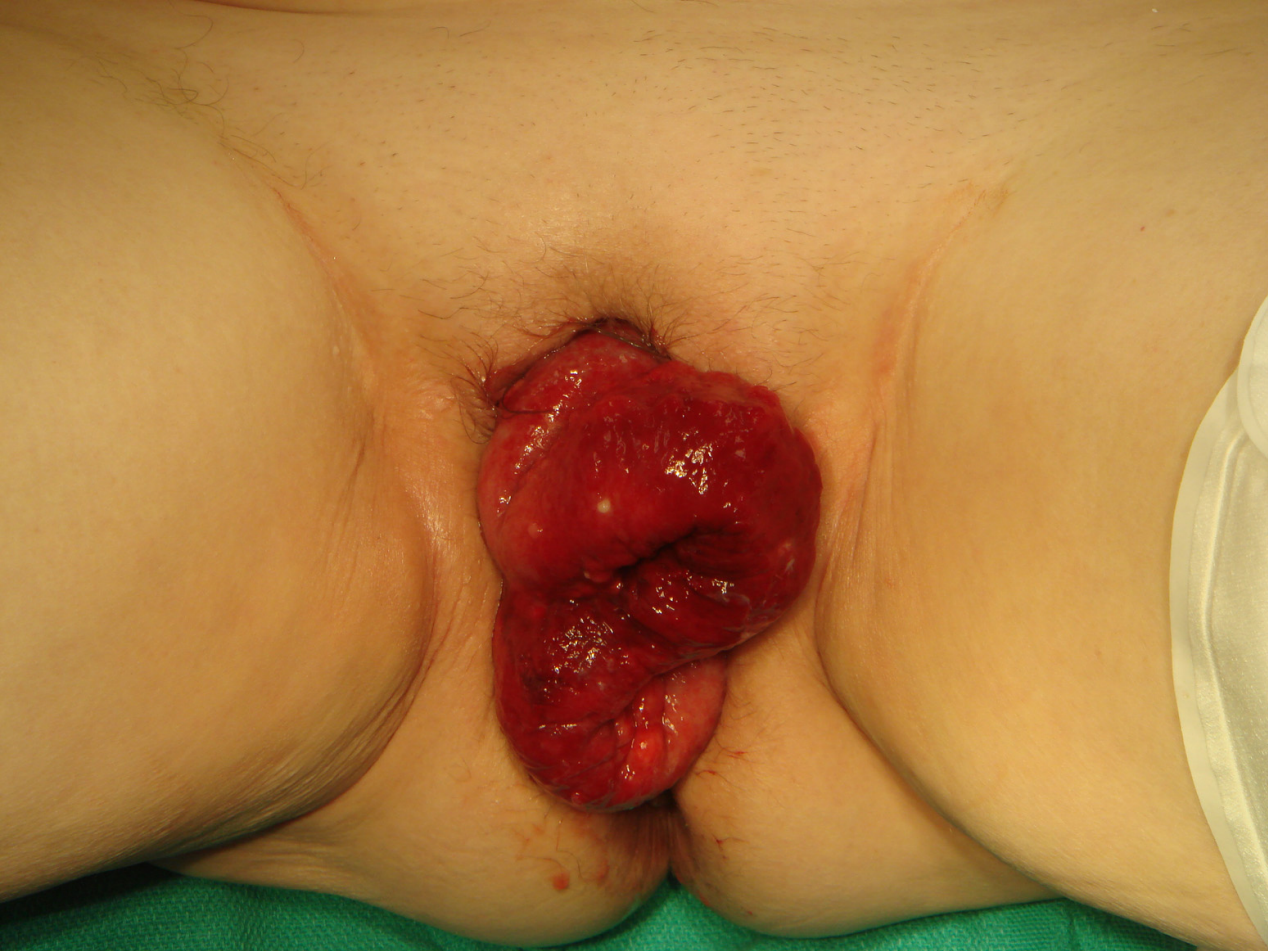
**Transvaginal evisceration of small bowel**.

On examination she was pleasant, appearing well and in no acute distress, with a temperature of 36.8 C°, pulse of 70, and blood pressure of 142/97 mmHg. Initial abdominal examination demonstrated no tenderness to palpation and no peritoneal signs. On genitourinary exam approximately 15 cm of small bowel protruded through the vaginal introitus (see Figure 1). The bowel exhibited peristaltic waves and was dark red in color. Manual reduction was attempted to reduce strangulation, but was unsuccessful because of the large amount of bowel present and to the patient's discomfort during the attempt. Intravenous morphine was administered for pain control. Sterile moist gauze was placed over the eviscerated bowel, and the gynecology department was immediately consulted for surgical management of the patient. The patient was given a bolus of 1 l normal saline and intravenous ertapenam and metronidazole to cover enteric organisms.

Laboratory data results included white blood cell count, 5.9 k/ul; hemoglobin, 13.1 g/dl; hematocrit, 38.6%; platelets, 287 k/ul; prothrombin time and partial thromboplastin time were normal. Sodium was 142 mEq/l; chloride, 109 mEq/l; potassium, 3.8 mEq/l; bicarbonate 23 mEq/l; glucose, 123 mg/dl; blood urea nitrogen, 17 mg/dl; creatinine, 0.7 mg/dl; calcium 9.3 mg/dl. The electrocardiogram showed normal sinus rhythm, with left atrial enlargement.

While awaiting surgical consultation and 45 min after being placed in a room, the patient developed relative hypotension; her blood pressure decreased from 183/108 to 107/63 mmHg. She became less responsive and experienced rigors. Repeat abdominal exam showed diffuse abdominal tenderness with peritoneal signs that rapidly progressed to frank peritonitis. The herniated bowel had become dark and dusky. A second large bore IV was placed, and aggressive fluid resuscitation with 2 l normal saline was initiated. Her bed was placed in the Trendelenburg position to reduce tension on the eviscerated bowel, and preparations were made to intubate the patient because of her rapid decline. The fluid resuscitation was successful in improving her mental status and blood pressure, and she did not require intubation. She was transported quickly to the operating room for repair by both gynecology and general surgery physicians. General surgery resected approximately 20 cm of the distal ileum, which they noted to be inflamed and thickened with two areas of ischemia. This was followed by resection and repair of the vaginal cuff by gynecologic surgery. The patient was discharged from the hospital 6 days later in improved condition.

## Discussion

Since the first account of transvaginal evisceration was reported in the English literature in 1907, fewer than 100 cases have been reported [2-5]. This literature is predominantly in the fields of obstetrics and gynecology, and only two articles on this topic have been presented in journals specific to emergency medicine [6,7]. Not all cases of evisceration are as obvious as our patient, so patients with similar complaints without obvious bowel herniation should undergo a pelvic exam to assess for the presence of cuff defect and transvaginal evisceration. Physicians should recognize this condition largely occurs among patients who have undergone menopause or hysterectomy. A large case series from a single institution concluded that this diagnosis should be considered in any woman presenting with acute vaginal bleeding and pelvic pain, especially postmenopausal women with a history of prolapse and pelvic surgery [4]. The median time to evisceration after pelvic surgery is 20 months [2]. Most patients report sudden onset of abdominal pain, a mass protruding from the vagina, vaginal bleeding, nausea, or vaginal discharge [2]. Despite the potential for significant morbidity, most patients with transvaginal evisceration present with acute, but subtle symptoms and rarely display evidence of peritonitis on presentation [2]. The combination of subtle presenting complaints and the need for a pelvic exam to obtain the clinical diagnosis predispose these patients to delayed diagnosis.

Emergency department management of a patient with transvaginal evisceration was discussed by Guttman et al. in 1990 and parallels the approach taken in our case: stabilization including fluid therapy, wrapping the exposed bowel in saline-soaked gauze, and administration of broad-spectrum antibiotics that cover gastrointestinal flora in preparation for immediate surgical repair. If the protruding intestines appear viable with obvious peristalsis and pink coloration, sterile saline irrigation of the exposed bowel and manual replacement through the vaginal cuff should be attempted [2,7,8]. If the reduction is successful, the patient may undergo primary transvaginal cuff repair without laparotomy. If the bowel cannot be reduced, the patient should proceed directly to laparotomy. Both gynecologic and general surgery should be consulted since definitive treatment may require bowel resection as well as vaginal cuff repair. The combined approach allows for more thorough inspection of the bowel and resection of ischemic sections as necessary. Gynecology can then proceed with vaginal cuff repair.

Transvaginal eviscerations have resulted in two reported deaths; therefore, it is important to include it in the differential diagnosis of a woman presenting with abdominal pain, perform a complete physical exam, and treat an evisceration promptly [7]. Laboratory values including CBC and electrolytes may be helpful in identifying patients with unanticipated anemia or electrolyte abnormalities; however, no laboratory results are specific in identifying patients with transvaginal evisceration. Serum lactate may predict the presence of ischemic bowel, but a normal serum lactate does not preclude the need for surgical intervention. Early recognition as well as management is the cornerstone of reducing morbidity and mortality associated with this subtle but serious condition.

According to the National Hospital Ambulatory Medical Care Survey data, abdominal pain is the most common presentation to US emergency departments [9]. Emergency department overcrowding has been associated with delayed care in patients with severe pain [10,11]. This case highlights the challenges of appropriately triaging patients whose complaints are not easily assessed in triage. According to most triage classifications, abdominal pain represents an urgent condition that requires prompt care, but will not cause life or limb threat if not treated for several hours. Abdominal pain is the most frequent presenting chief complaint to US emergency departments and represents a broad spectrum of disease states ranging from benign to life-threatening etiologies. The etiology of our patient's pain was easily recognized in the treatment area, but her well appearance and the vagueness of her symptoms led the triage nurse to classify this patient as urgent instead of emergent. In many over-utilized US EDs, this non-emergent classification of patients with transvaginal evisceration could lead to significant delays in diagnosis. Without prompt attention to her worsening pain, this patient could have experienced an adverse outcome. Triage nurses should be educated to recognize benign-appearing presentations of life-threatening conditions. From 1997 through 2006, the number of ED visits increased from 94.9 million to 119.2 million, representing an increase of 24 percent [9]. A national survey of ED directors defined ED crowding as waiting greater than 1 h to see a physician; this wait is more likely to result in adverse outcomes [12]. Studies have found disagreement exists among health care professionals about the urgent needs of emergency department patients even when using the same criteria [13-15]. EM physicians should consider alternative triage methods for patients with sensitive complaints possibly representing life-threatening emergencies that can't be appropriately assessed in a semi-private triage area to reduce the morbidity and mortality associated with delayed diagnosis. Triage nurses should be educated on the importance of regular reassessment of vital signs since patients who appear stable on initial presentation may rapidly decompensate.

## Conclusion

This case demonstrates the need to perform a thorough physical exam on all patients with abdominal pain and details the management of vaginal evisceration. This case also highlights the difficulty of appropriate triage for patients with complaints not easily assessed in triage. In an era of emergency department crowding, emergency physicians should reevaluate nursing education on triaging abdominal pain to prevent delays in caring for well-appearing patients who have underlying life-threatening illnesses.

## Consent

Written informed consent was obtained from the patient for publication of this case report and any accompanying images. A copy of the written consent is available for review by the Editor-in-Chief of this journal.

## Competing interests

The authors declare that they have no competing interests.

## Authors' contributions

KC treated the patient in the ED and was involved in drafting the manuscript. LL cared for the patient in the ED and was involved in drafting the manuscript, major revisions, and editing of the manuscript. LP was involved in critical editing and revisions of the manuscript. LL and LP participated in its design and coordination. All authors read and approved the final manuscript.

## Authors' information

LL, Assistant Professor, Department of Emergency Medicine, Brody School of Medicine at East Carolina University, Greenville, NC

LP, Assistant Professor, Department of Emergency Medicine, Brody School of Medicine at East Carolina University, Greenville, NC

KC, Emergency Medicine, Johnston Memorial Hospital, Abingdon, VA
